# Agronomic and chemical performance of field‐grown tobacco engineered for triterpene and methylated triterpene metabolism

**DOI:** 10.1111/pbi.12855

**Published:** 2018-01-03

**Authors:** Zuodong Jiang, Chase Kempinski, Santosh Kumar, Scott Kinison, Kristin Linscott, Eric Nybo, Sarah Janze, Connie Wood, Joe Chappell

**Affiliations:** ^1^ Plant Biology Program University of Kentucky Lexington KY USA; ^2^ Department of Pharmaceutical Sciences University of Kentucky Lexington KY USA; ^3^ Molecular and Cellular Biochemistry University of Kentucky Lexington KY USA; ^4^ Department of Statistics University of Kentucky Lexington KY USA

**Keywords:** terpene engineering, field trials, chemical performance, agronomic performance

## Abstract

Squalene is a linear intermediate to nearly all classes of triterpenes and sterols and is itself highly valued for its use in wide range of industrial applications. Another unique linear triterpene is botryococcene and its methylated derivatives generated by the alga *Botryococcus braunii* race B, which are progenitors to fossil fuel deposits. Production of these linear triterpenes was previously engineered into transgenic tobacco by introducing the key steps of triterpene metabolism into the particular subcellular compartments. In this study, the agronomic characteristics (height, biomass accumulation, leaf area), the photosynthetic capacity (photosynthesis rate, conductance, internal CO_2_ levels) and triterpene content of select lines grown under field conditions were evaluated for three consecutive growing seasons. We observed that transgenic lines targeting enzymes to the chloroplasts accumulated 50–150 times more squalene than the lines targeting the enzymes to the cytoplasm, without compromising growth or photosynthesis. We also found that the transgenic lines directing botryococcene metabolism to the chloroplast accumulated 10‐ to 33‐fold greater levels than the lines where the same enzymes were targeted to in the cytoplasm. However, growth of these high botryococcene accumulators was highly compromised, yet their photosynthesis rates remained unaffected. In addition, in the transgenic lines targeting a triterpene methyltransferase (TMT) to the chloroplasts of high squalene accumulators, 55%–65% of total squalene was methylated, whereas in the lines expressing a TMT in the cytoplasm, only 6%–13% of squalene was methylated. The growth of these methylated triterpene‐accumulating lines was more compromised than that of nonmethylated squalene lines.

## Introduction

Tobacco is an annually grown herbaceous plant that produces over 2500 compounds including terpenes, alkaloids, flavonoids and anthocyanins (Nugroho and Verpoorte, 2002). It has been grown primarily for its use in smoke and smokeless consumer products that have abundant amounts of nicotine, nornicotine, anabasine and anatabine, thought important for its consumer preference (Siminszky *et al*., 2005). Tobacco has received much more attention recently because genetically enhanced tobacco has been suggested as an alternative platform for pharmaceuticals and biofuel production (Wu *et al*., 2012). For example, vaccines and immunotherapies produced in plants such as tobacco have been validated as alternative production platforms, potentially mitigating production shortages, costs and biohazards associated with mammalian cell culture systems (Ma *et al*., 2015; Yusibov *et al*., 2011). As an industrial biomass crop, tobacco can yield up to 170 tons per hectare of green biomass. When coupled with various genetic engineering approaches, transgenic lines can provide up to 6% of their dry weight as lipids and fatty acids suitable for biofuel and biodiesel uses, rivalling oil seed production capacities (Andrianov *et al*., 2010).

**Figure 1:**
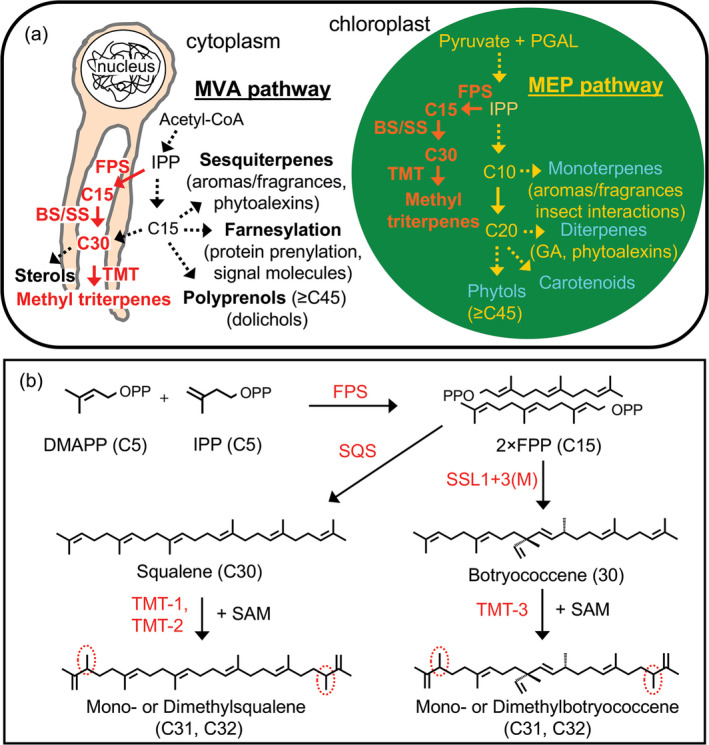
Schematic outline of the mevalonate (MVA) and methylerythritol phosphate (MEP) pathways operating in the cytoplasm and chloroplast compartments, respectively, and conceptual strategies to divert carbon flux from these two pathways for the biosynthesis of triterpene (C30) by heterologous expression of an avian FPP synthase (FPS) and botryococcene synthase (BS) or squalene synthase (SS), which can be further methylated by triterpene methyltransferases (TMTs) (a). A depiction of triterpene and methylated triterpene biosynthesis (b).

Engineering high‐level production of terpenes in tobacco has been achieved using a variety of strategies generating large amounts of sesquiterpene, monoterpene and triterpene products (Kempinski *et al*., 2016; Lange and Ahkami, 2013). One of the most successful approaches has relied upon the diversion of carbon flux from the MEP pathway by overexpression and targeting an avian farnesyl diphosphate (FPP) synthase (FPS) gene along with a heterologous terpene synthase gene to the chloroplast compartment (Figure 1a; Wu *et al*., 2006). Engineering FPS to create a plastidic FPP pool was key to the success of this strategy because FPP biosynthesis is lacking in the plastid and hence is not subject to any known innate regulation in the chloroplasts (Kappers *et al*., 2005). In contrast, cytosolic biosynthesis of FPP is highly regulated (Gardner and Hampton, 1999; Janowski *et al*., 1996). Thus, the strategy derives from the putative unlimited supply of IPP/DMAPP that can be diverted from the MEP pathway by the action of FPS to yield novel pools of plastidic FPP. These plastidic pools can then be utilized by FPP‐dependent terpene synthases targeted to the chloroplasts for production and accumulation of novel terpene(s) compounds (Wu *et al*., 2006, 2012).

This strategy has made it possible to envision production of high‐valued triterpene products including squalene and organism‐specific botryococcene and methylated triterpenes generated solely by the green algae *Botryococcus braunii*, race B. All of these triterpenes could be considered as alternative sources for biofuels, vaccine adjuvants, emollients and other industrial applications (Figure 1b; Niehaus *et al*., 2011). The genes encoding for the unique enzymes responsible for botryococcene and methylated triterpene biosynthesis in *Botryococcus* were previously characterized by Niehaus *et al*. (2011). In short, a squalene synthase‐like enzyme, SSL‐1 catalyses the condensation of two FPP molecules into an intermediate product, presqualene diphosphate, which is then converted by another squalene synthase‐like enzyme, SSL‐3, to form botryococcene (Figure 1b; Niehaus *et al*., 2011). A series of triterpene methyltransferases (TMTs) were also found that could methylate C30 triterpene (squalene or botryococcene) into their methylated derivatives (Figure 1b; Niehaus *et al*., 2012). Triterpene methyltransferase 1 (TMT‐1) and triterpene methyltransferase 2 (TMT‐2) prefer squalene C30 as their substrate for the generation of mono‐ (C31) or dimethylated (C32) squalene, while TMT‐3 prefers botryococcene as its substrate for the biosynthesis of mono‐ (C31) or dimethylated (C32) botryococcene (Figure 1b; Niehaus *et al*., 2012). These methylated derivatives are highly desirable for fuels due to their high energy content and hydrocracking to high octane forms (Hillen *et al*., 1982; Metzger, 1985; Metzger *et al*., 1988).

Use of plants as a platform for triterpene production purposes is promising because plants can utilize photosynthesis to directly convert solar energy and CO_2_ into high‐value products and because of the ready scalability of plant propagation from glasshouse to large‐scale field operations at relatively low costs. Transgenic tobacco engineered for high‐level accumulation of squalene (C30), botryococcene (C30) and their methylated derivatives (C31–C32) were described previously (Jiang *et al*., 2016; Wu *et al*., 2012). However, the impact such biochemical/metabolic engineering efforts might have on the overall physiology and yield of triterpene oils in field‐grown plants was unknown. Therefore, to gain a better appreciation for the robustness of the triterpene accumulation trait and its impact on overall growth performance, an array of transgenic lines were grown in field trials for three consecutive years and evaluated for their agronomic performance and triterpene content.

## Results

### Transgenic lines and field analyses

**Figure 2:**
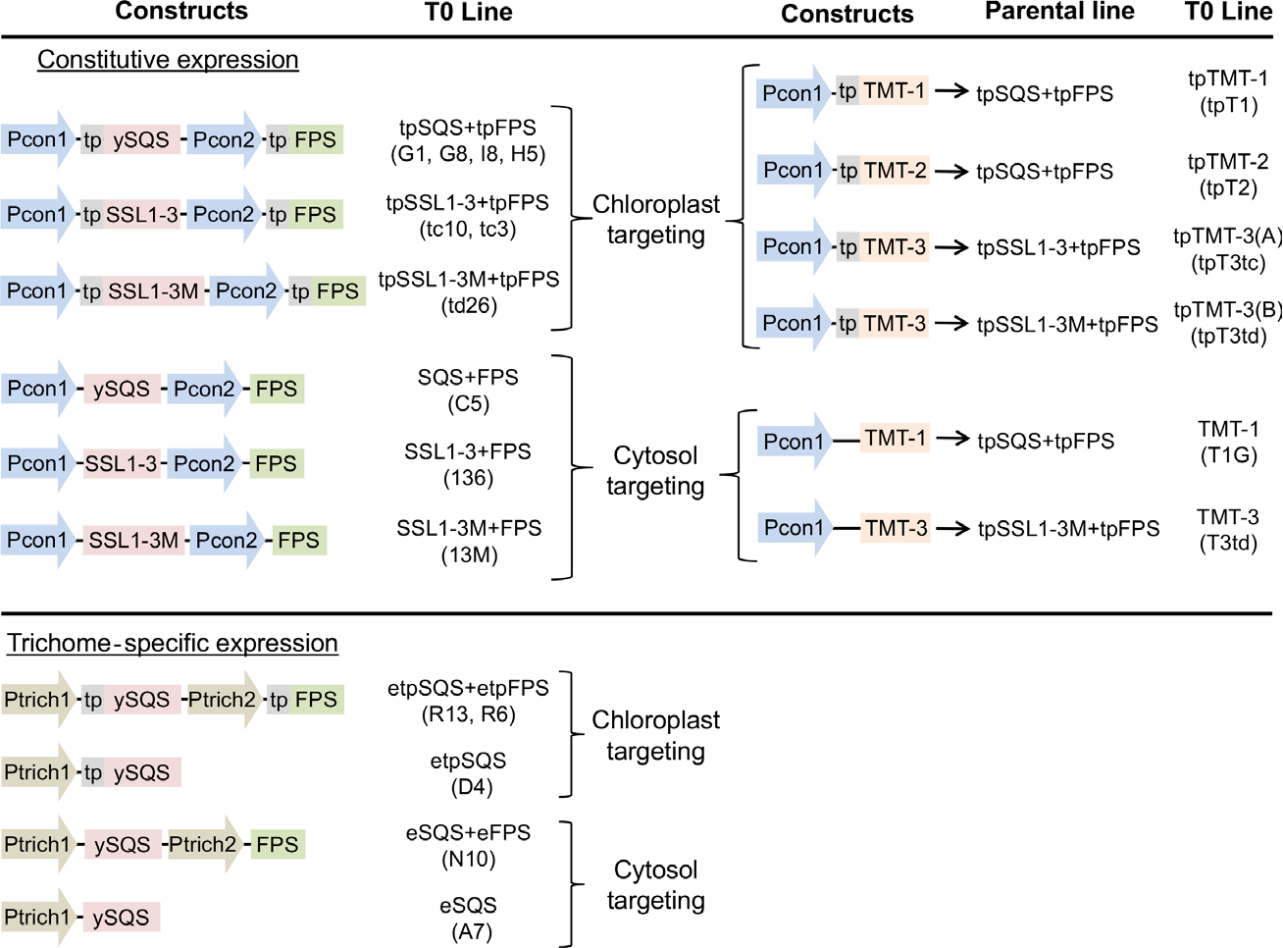
Gene constructs used to engineer constitutive or trichome‐specific expression, as well as targeting triterpene metabolism to the cytosol or plastid (tp) compartments used in this study. Construct design was based on the work previously described by Wu *et al*. (2006). Gene constructs consisted of a truncated form of the yeast squalene synthase (ySQS) gene (ERG9, GenBank accession NM 001179321) (Zhang *et al*., 1993), fused squalene synthase‐like genes 1 and 3 (SSL1‐3) (HQ585058, HQ585060) from *Botryococcus braunii* creating a chimeric botryococcene synthase, including a carboxy‐terminal membrane spanning domain from the *B. braunii* squalene synthase (SSL1‐3M) (Niehaus *et al*., 2011), and the avian farnesyl diphosphate synthase (FPS) gene (P08836) (Tarshis *et al*., 1994). The truncated SQS was created by PCR amplifying the yeast SQS mRNA from its start codon to nucleotide 1260, thus deleting the DNA encoding for the carboxy‐terminal 24 amino acids. These carboxy‐terminal amino acids are predicted to tether the SQS protein to membranes, and the deletion of these amino acids creates a functionally soluble enzyme (Zhang *et al*., 1993). The ySQS, SSL1‐3 and FPS genes were inserted downstream of strong constitutive promoters Pcon1 (35S cauliflower mosaic viral promoter) (Benfey and Chua, 1990); Pcon2 (cassava vein mosaic viral promoter) (Verdaguer *et al*., 1998), or trichome‐specific promoters Ptrich1 (cembratrien‐ol synthase promoter, Pcbt) (Ennajdaoui *et al*., 2010) or Ptrich2 (cembratrien‐ol hydroxylase promoter, Pcyp16) (Wang *et al*., 2002). The Pcbt and Pcyp16 promoters were further augmented with duplicated CAMV 35S enhancer elements (Benfey *et al*., 1990) fused to the 5′ termini of the promoters (i.e. etpSQS). Where indicated, a plastid targeting signal sequence (tp) encoding for the first 58 amino acids of the Arabidopsis RuBisCO small subunit gene (NM23202) (Lee *et al*., 2006) was fused onto the 5′ end of the respective genes. Methylated triterpene‐accumulating lines were generated by transforming squalene‐accumulating or botryococcene‐accumulating parental lines with one of three methyltransferase genes (TMT‐1, 2 or 3) (Niehaus *et al*., 2012). The specific plant lines field tested are noted in parentheses.

Independent T0 transgenic lines expressing squalene synthase (SQS) with FPS targeted to the chloroplasts or the cytoplasm under the direction of constitutive (CaMV35S (Benfey and Chua, 1990) and CV35S cassava mosaic virus (Verdaguer *et al*., 1998)) or trichome‐specific promoters (cbt1, cembratrien‐ol synthase (Ennajdaoui *et al*., 2010) and CYP16 cytochrome P450 71D16 (Wang *et al*., 2002)) were generated by transforming each indicated construct into KY 1068 cultivars as described previously (Figure 2, Wu *et al*., 2012). The botryococcene‐accumulating lines expressing plastidic or cytosolic botryococcene synthase (SSL1‐3(M)) with FPS were also generated in the study as described in Jiang *et al*. (2016). The parental lines (T0) were then self‐propagated and the T1 and T2 generations tested for homozygosity or heterozygosity of the transgene cassette by antibiotic‐based (hygromycin) segregation screens and chemical profiling (hexane extraction followed by GC‐MS). T1 lines constitutively expressing plastidic‐targeted botryococcene synthase (SSL1‐3(M)) with FPS, or homozygous T2 lines constitutively expressing plastidic‐targeted SQS, were engineered with TMTs targeted to the cytoplasm or the chloroplast, yielding lines with high levels of triterpene biosynthesis targeted to the plastid compartment with various combinations of methyltransferases targeted to either the plastid or cytoplasmic compartments (Figure 2; Jiang *et al*., 2016). T1 generation, heterozygous lines expressing the TMTs were chosen for field studies in 2013 and 2014.

Agronomic characteristics (height, biomass accumulation, leaf area) were monitored throughout a typical field season of approximately 90 days from late May to early September, but the final measurements at the termination of each field study are used here to simplify the comparisons. Likewise, measurements of CO_2_ fixation (photosynthesis rate), transpiration (conductance) and internal CO_2_ levels (Ci) were taken at various times during the field trials, but the final determination at ~60 days after planting is used to compare physiological measurements of fitness. The triterpene content was determined for the uppermost, fully expanded leaf of 6–9 plants for each transgenic line grown in three replicate rows at two growing stages: green tissue (45–50 days) and senesced tissue (80–100 days, after topping) for three growing seasons. Triterpene content was quantified by GC‐FID, GC‐MS analyses (Jiang *et al*., 2016; Wu *et al*., 2012).

### Overall field performance

In general, the growth characteristics for the lines grown for three consecutive years were significantly greater in 2013 than for 2012 and 2014 (see Tables 1, 2, 3, 4, 5). For instance, control, wild‐type (WT) plants grown in 2013 exhibited biomass (leaf weight and total weight) and leaf area measurements 1.4 to 2.1 times greater than those in 2012 and 2014 (Table 1). Rainfall was significantly greater in 2013 than in 2012 and 2014 (Table 2).

**Table 1:** Comparison of the growth characteristics and squalene accumulation by control, wild‐type (WT) plants grown for three seasons (2012–2014)

WT year	Height (metres)	Leaf weight (kg)	Total weight (kg)	Leaf area (cm^2^)	Photosynthesis (μmol CO_2_/m^2^/s)	Conductance (mol H_2_O/m^2^/s)	Ci (μmol CO_2_/mol air)	Squalene (μg/g fresh weight)
2012 Group	1.35 + 0.12 A	1.71 + 0.25 A	2.58 + 0.33 A	20 604 + 2282 A	21.98 + 3.76 A	0.42 + 0.15 A	227 + 17.7 A	7.08 + 1.01 A
2013 Group	1.74 + 0.12 B	3.14 + 0.48 B	5.46 + 0.52 B	38 302 + 5660 B	18.2 + 1.17 B	0.28 + 0.05 B	202.2 + 12.0 B	7.71 + 3.21 A
2014 Group	1.62 + 0.21 B	2.23 + 0.64 C	3.61 + 0.79 C	26 380 + 4421 C	22.6 + 1.26 A	0.42 + 0.06 A	206.1 + 15.8 B	4.38 + 5.5 A

Each value represents the average of the biological replicates within each group ±SD. The value within each different group (A, B or C) indicates a significant difference from each other group as determined by a *t*‐test (*P* < 0.05).

**Table 2:** Precipitation (inches) at the field trial location for 2012–2014

Year\Month	May	June	July	August	Total growing season
2012	4.25	1.5	5.54	3.17	14.46
2013	4.44	6.23	6.42	4.14	21.23
2014	3.65	3.98	3.67	5.43	16.73

Data collected from University of Kentucky Ag Weather Center.

**Table 3:** Squalene production and agronomic performance of transgenic tobacco engineered for squalene biosynthesis in 2012 and 2013

T0 line	T2 line	Squalene accumulation (μg/g fresh weight)	Leaf weight (kg)	Total weight (kg)	Height (m)	Leaf area (cm^2^)
		Green tissue	Senesced tissue	Biomass		
Year (2012)							
Constitutive expression							
tpSQS + tpFPS	G1	666.5 ± 186.7*	280.1 ± 65.1*	1.69 ± 0.34	2.49 ± 0.47	1.30 ± 0.09	22 636 ± 4710
tpSQS + tpFPS	G8	609.3 ± 143.7*	242.4 ± 158.3*	1.49 ± 0.26	2.24 ± 0.34	1.27 ± 0.15	21 958 ± 2895
tpSQS + tpFPS	I8	572.2 ± 97.1*	209.0 ± 106.6*	1.30 ± 0.38*	1.94 ± 0.56*	1.26 ± 0.16	17 803 ± 5379
tpSQS + tpFPS	H5	419.3 ± 258.2*	132.5 ± 45.0*	1.25 ± 0.47*	1.82 ± 0.70*	1.11 ± 0.33*	16 366 ± 6724
SQS + FPS	C5	7.7 ± 0.9	8.7 ± 1.6	1.06 ± 0.24*	1.68 ± 0.28*	1.32 ± 0.14	14 043 ± 3373*
Trichome expression							
etpSQS + etpFPS	R13+	587.8 ± 180.8*	110.7 ± 44.4*	0.91 ± 0.22*	1.3 ± 0.33*	0.94 ± 0.15*	13 019 ± 3135*
	R13−	14.0 ± 15.5	7.72 ± 8.62	1.69 ± 0.31	2.55 ± 0.47	1.37 ± 0.17	19 597 ± 1875
etpSQS	D4	26.2 ± 7.7	21.7 ± 8.33	1.94 ± 0.37	3.03 ± 0.56	1.51 ± 0.15	23 727 ± 4772
eSQS	A7	8.2 ± 1.8	8.78 ± 2.27	1.53 ± 0.26	2.43 ± 0.48	1.49 ± 0.17	19 220 ± 3595
eSQS + eFPS	N10	7.9 ± 1.4	7.19 ± 2.13	1.46 ± 0.30	2.25 ± 0.50	1.38 ± 0.17	17 111 ± 4734
Nontransgenic control							
Wild type	WT	7.9 ± 1.0	1.70 ± 0.89	1.71 ± 0.25	2.58 ± 0.33	1.35 ± 0.12	20 604 ± 2282
Year (2013)							
Constitutive expression							
tpSQS + tpFPS	G1	377.9 ± 116.0*	305.8 ± 146.9*	3.07 ± 0.40	4.87 ± 0.61	1.57 ± 0.11	33 339 ± 5766
tpSQS + tpFPS	G8	274.9 ± 66.3*	314.7 ± 125.6*	2.51 ± 0.22*	4.0 ± 0.46*	1.54 ± 0.14	31 013 ± 5550*
tpSQS + tpFPS	I8	222.8 ± 76.5*	244.0 ± 97.3*	2.83 ± 0.34	4.56 ± 0.49*	1.61 ± 0.09	33 295 ± 3712
tpSQS + tpFPS	H5	309.1 ± 79.3*	132.7 ± 125.3*	1.44 ± 0.31*	2.07 ± 0.43	1.1 ± 0.16*	19 670 ± 5813*
SQS + FPS	C5	2.5 ± 1.8	14.9 ± 5.9	1.76 ± 0.20*	3.37 ± 0.27*	1.8 ± 0.12	22 831 ± 1974*
Trichome expression							
etpSQS + etpFPS	R13+	247.5 ± 97.7*	178.9 ± 89.6*	1.76 ± 0.20*	2.79 ± 0.36*	1.41 ± 0.21*	24 816 ± 2692*
	R13−	1.4 ± 0.5	12.5 ± 9.5	2.95 ± 0.70	5.28 ± 1.16	1.63 ± 0.08	35 312 ± 7498
etpSQS + etpFPS	R6	160.9 ± 68.5*	215.9 ± 140.2*	1.92 ± 0.31*	2.87 ± 0.57*	1.29 ± 0.35*	25 918 ± 5891*
etpSQS	D4	11.6 ± 4.3	15.8 ± 18.1	2.68 ± 0.42*	4.79 ± 0.67	1.7 ± 0.23	33 019 ± 4733
eSQS	A7	6.5 ± 3.3	3.8 ± 0.6	2.34 ± 0.31*	4.25 ± 0.44*	1.75 ± 0.17	29 420 ± 3080*
eSQS + eFPS	N10	1.9 ± 2.2	6.2 ± 5.8	2.30 ± 0.36*	4.10 ± 0.59*	1.73 ± 0.26	26 945 ± 4670*
Nontransgenic control							
Wild type	WT	7.7 ± 3.2	9.7 ± 2.2	3.14 ± 0.48	5.46 ± 0.52	1.74 ± 0.12	38 302 ± 5660

The values shown are the averages of determinations of 6–9 individual plants from three independent rows. Each value represents the average of the biological replicates within each group ±SD. An asterisk for a transgenic group indicates a significant difference from wild‐type control group as determined by LSD *t*‐test (*P* < 0.05). Schematics for constructs and abbreviations are defined in Figure 2.

**Table 4:** Analysis of botryococcene accumulation and agronomic performance of transgenic tobacco engineered for botryococcene biosynthesis in 2013 and 2014

T0 line	T1 line	Botryococcene (μg/g fresh weight)	Leaf weight (kg)	Total weight (kg)	Height (m)	Leaf area (cm^2^)
		Green tissue	Senesced tissue	Biomass		
Year (2013)							
tpSSL1 + 3M	td26+	119.0 ± 46.6*	106.6 ± 32.0*	1.87 ± 0.26*	3.0 ± 0.28*	1.31 ± 0.22*	22 650 ± 4921*
	td26−	0	0	2.55 ± 0.68*	4.60 ± 1.13*	**1.67 ± 0.10**	**35 197 ± 9078**
tpSSL1 + 3	tc10+	260.8 ± 164.6*	221.9 ± 162.2*	2.03 ± 0.62*	3.14 ± 0.99*	1.28 ± 0.21*	26 137 ± 6106*
	tc10−	0	0	**2.76 ± 0.16**	**4.80 ± 0.31**	**1.80 ± 0.20**	30 275 ± 7410*
Wild type	WT	0	0	3.14 ± 0.48	5.46 ± 0.52	1.74 ± 0.12	38 302 ± 5660
Year (2014)							
tpSSL1 + 3M	td26+	105.4 ± 26.0*	42.2 ± 25.3*	**1.79 ± 0.19**	2.70 ± 0.26*	1.23 ± 0.35*	**24 014 ± 5088**
	td26−	0	0	2.83 ± 0.57*	4.67 ± 1.45*	**1.56 ± 0.31**	**30 456 ± 8920**
tpSSL1 + 3	tc10+	259.0 ± 106.7*	197.6 ± 196.0*	1.51 ± 0.50*	2.40 ± 0.72*	1.19 ± 0.12*	15 332 ± 3592*
	tc10−	0	0	**2.47 ± 0.36**	**4.12 ± 0.63**	**1.49 ± 0.18**	**29 797 ± 5761**
tpSSL1 + 3	tc3+	217.2 ± 130.1*	159.9 ± 115.2*	1.40 ± 0.32*	2.17 ± 0.47*	1.16 ± 0.16*	17 410 ± 4605*
	tc3−	0	0	**2.75 ± 0.30**	4.39 ± 0.36*	**1.58 ± 0.22**	32 707 ± 2741*
SSL1 + 3M	13M+	9.9 ± 5.1	4.2 ± 4.5	**1.80 ± 0.30**	**3.28 ± 0.89**	1.47 ± 0.14	22 051 ± 1534
	13M−	0	0	**2.43 ± 0.71**	4.46 ± 1.02*	1.50 ± 0.09	26 153 ± 7670
SSL1 + 3	136+	11.0 ± 13.5	6.0 ± 5.1	2.21 ± 0.58	4.1 ± 0.73	1.42 ± 0.14*	28 670 ± 7736
	136−	0	0	2.12 ± 0.46	3.54 ± 0.63	1.44 ± 0.23*	30 930 ± 612
Wild type	WT	0	0	2.23 ± 0.64	3.61 ± 0.79	1.62 ± 0.21	26 380 ± 4421

The values shown are the averages of determinations of 6–9 individual plants from three independent rows. Each value represents the average of the biological replicates within each group ±SD. An asterisk for a transgenic group indicates a significant difference from wild‐type control group, and bold values denote statistically significant differences between heterozygous sibling populations segregating for botryococcene metabolism (+) or not (−) as determined by LSD *t*‐test (*P* < 0.05). Schematics for constructs and abbreviations are defined in Figure 2.

**Table 5:** Analysis of triterpene accumulation and agronomic performance of transgenic tobacco engineered for methylated triterpene biosynthesis in 2014

T0 line	T1 line	Total triterpene (μg/g fresh weight)	Methyl triterpene (μg/g fresh weight)	Conversion (methylated/total)	Leaf weight (kg)	Total weight (kg)	Height (m)	Leaf area (cm^2^)
		Green tissue			Biomass			
Year 2014								
tpTMT‐1	tpT1+	496.8 ± 209.0*	278.9 ± 125.3*	0.57 ± 0.11	1.31 ± 0.24*	2.04 ± 0.40*	1.19 ± 0.23*	17 022 ± 3048*
	tpT1−	379.5 ± 109.6*	0	0	**1.90 ± 0.32**	2.81 ± 0.55*	1.26 ± 7.4*	19 679 ± 3942*
tpTMT‐2	tpT2+	326.0 ± 81.4*	230.8 ± 53.1*	0.71 ± 0.06	1.45 ± 0.43*	2.22 ± 0.49*	1.13 ± 0.13*	18 447 ± 5302*
	tpT2−	165.2 ± 52.2*	0	0	**1.98 ± 0.60**	**3.13 ± 1.02**	1.29 ± 0.12*	22 946 ± 2539
TMT‐1	T1G+	225.8 ± 75.5*	13.7 ± 4.9	0.06 ± 0.02	1.80 ± 0.53	2.70 ± 0.56*	1.14 ± 0.12*	23 790 ± 4516
	T1G−	199.5 ± 103.8*	0	0	2.00 ± 0.38	3.00 ± 0.52	1.10 ± 0.59*	25 126 ± 5281
tpTMT‐3(A)	tpT3tc+	292.6 ± 193.5*	196 ± 111*	0.73 ± 0.12	1.35 ± 0.28*	**2.19 **± 0.49*	1.10 ± 0.11*	21 412 ± 5844
	tpT3tc−	266.4 ± 135.8*	0	0	**1.98 ± 0.59**	**3.11 ± 0.82**	1.12 ± 0.10*	22 423 ± 4549
tpTMT‐3(B)	tpT3td+	190.3 ± 45.9*	116.6 ± 41.0*	0.62 ± 0.19	1.88 ± 0.76	2.78 ± 1.22*	1.04 ± 0.31*	26 229 ± 9417
	tpT3td−	165.9 ± 53.0*		0	2.11 ± 0.50	3.24 ± 0.83	1.17 ± 0.12*	28 090 ± 2798
TMT‐3	T3td+	181.8 ± 65.4*	21.8 ± 9.1	0.13 ± 0.05	1.42 ± 0.33*	2.03 ± 0.41*	0.91 ± 0.1*	17 818 ± 2426*
	T3td−	200.6 ± 83.4*	0	0	1.45 ± 0.22*	2.10 ± 0.27*	1.01 ± 0.18*	20 275 ± 2688
Wild type	WT	4.38 ± 5.5	0	0	2.23 ± 0.64	3.61 ± 0.79	1.62 ± 0.21	26 380 ± 4421

The values shown are the averages of determinations of 6–9 individual plants from three independent rows. Each value represents the average of the biological replicates within each group ±SD. An asterisk for a transgenic group indicates a significant difference from wild‐type control group, and bold values denote statistically significant differences between heterozygous sibling populations segregating for triterpene methylation metabolism (+) or not (−) as determined by LSD *t*‐test (*P* < 0.05). Schematics for constructs and abbreviations are defined in Figure 2.

### Analysis of squalene accumulation on agronomic performance

Among all the T2 homozygous transgenic lines under the direction of constitutive promoters, the G1 line directing squalene biosynthesis to the chloroplast accumulated the highest level of squalene, which was 94‐ and 49‐fold (for green tissue), 165‐ and 32‐fold (for senescent tissue) higher than that found in WT plants in 2012 and 2013, respectively. Squalene accumulation by line C5, which has constitutive squalene biosynthesis targeted to the cytoplasm, was much closer to that measured for the WT control line in green tissue and only modestly higher (5.12‐ and 1.54‐fold) in senescent tissue for 2012 and 2013. Independent T2 lines engineered with the same construct used in generating G1 (and its sibling from the same transgenic event, G8), including H5, and I8 also yielded significantly higher levels of squalene accumulation over that in WT plants and cytosolic engineered lines (Table 3). These lines also tended to accumulate twofold or more squalene in green versus senescing leaves in 2012, but a much less differential was observed for 2013. In addition, by comparing the squalene level in most of the transgenic lines grown at different growing stages, we found that squalene levels in green tissue were usually higher than what was measured in more senesced tissue (Table 3). For instance, line G1 accumulated the highest average level of squalene across years 2012–2013 at about 522 μg/g fresh weight in green tissue, which was about two times greater than that in senescent tissue (Table 3). This pattern of accumulation is unlike that of other tobacco‐specific metabolites such as nicotine, which accumulates predominantly during the maturing or senescent stage (Yoshida and Takahashi, 1961).

Interestingly, high squalene accumulation did not seem to affect growth parameters, as lines G1 and G8 accumulated more squalene than I8 and H5 but did not show reduced growth versus WT in 2012. Specifically, the G1 line exhibited 96% and 90% the height, 99% and 98% the total weight, 97% and 89% the leaf weight and 110% and 87% the leaf area of that for WT plants in 2012 and 2013, respectively (Table 3). In 2013, the other two high squalene‐accumulating lines G8 and I8 showed a modest, but not significant, growth reduction (but G8 did not show reduced growth in 2012; Table 3). A significant growth reduction in both 2012 and 2013 was found for the H5 line, a moderate squalene accumulator (Table 3).

Although all of these high squalene accumulators were generated using the same engineering constructs, their growth characteristics were far from identical. More importantly, these field results indicated that plastidic biosynthesis and accumulation of squalene did not correlate with a decrease in agronomic performance per se. Instead, the genetic changes resulting from the transformation process, which might include nonspecific, position‐dependent effects or epigenetic changes, may have contributed to growth reduction in some transgenic lines, such as H5. This notion was also supported by line C5 which accumulated only a low level of squalene, similar to WT plants, but exhibited a pronounced decrease in leaf biomass without a significant reduction in height (Table 3). A reduced number of squalene‐accumulating lines were grown in year 2014 because of limited field space.

### Analysis of squalene accumulation on agronomic performance of plants engineered for trichome‐specific biosynthesis

**Figure 3:**
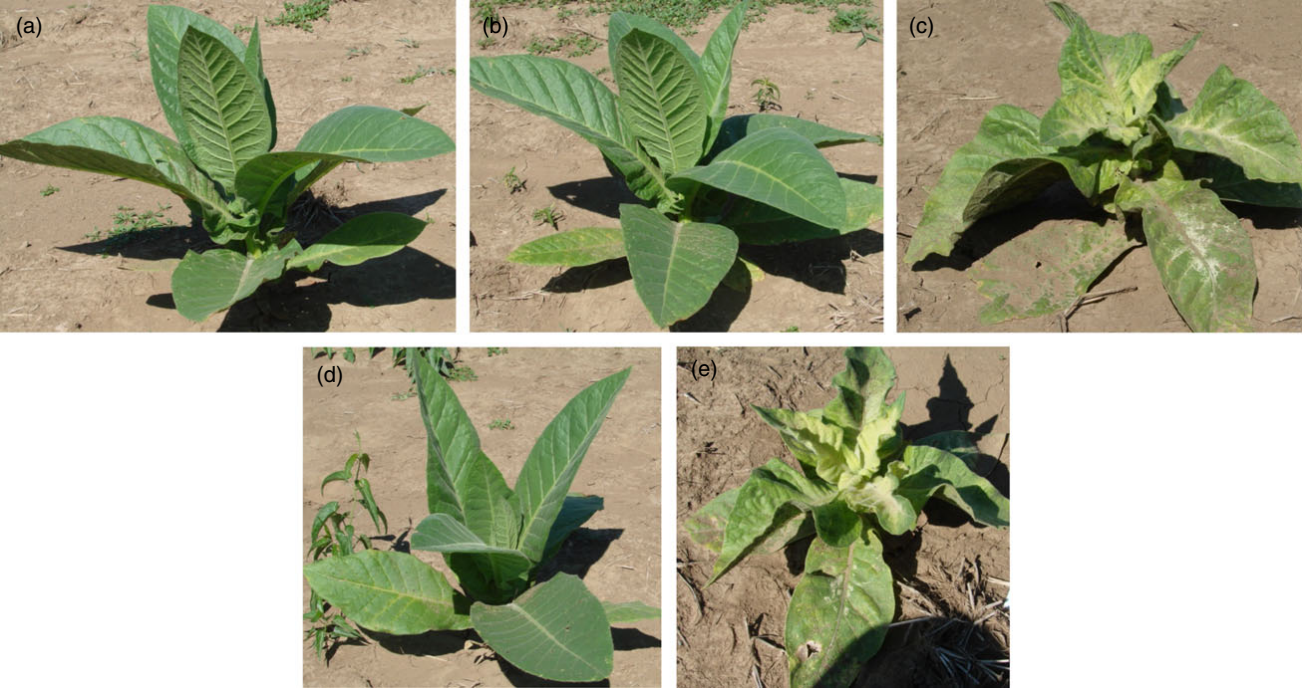
Phenotypes of field‐grown squalene‐accumulating lines directed by trichome‐specific gene promoters (c, line R6) and by strong constitutive gene promoters (b, line G1) relative to a wild‐type plant (a). Segregation of phenotypes within the progeny of line R13, engineered for squalene accumulation with the trichome‐specific promoters, was also readily apparent, R13− (d) not accumulating squalene, while R13+ (e) did.

The glasshouse‐grown T0 lines generated for trichome‐specific expression of SQS with FPS directed to the chloroplast compartment were previously determined to accumulate a high level of squalene and exhibit stunted growth and chlorotic symptoms (Wu *et al*., 2012), both of which were observed in T2 offspring for the heterozygous line R13 and homozygous line R6 grown under normal field conditions (Figure 3). For heterozygous R13 lines, those plants exhibiting stunted growth and chlorosis, as well as accumulating a significantly higher level of squalene, were considered to have inherited the transgenes and denoted as R13+. Those plants without visible abnormal phenotype and having squalene levels comparable to WT were considered to be siblings having lost the transgene during segregation and denoted as R13−. The squalene levels in green tissue from R13+ were 75‐ and 128‐fold greater than that for N10 that co‐expressed SQS and FPS targeted the cytoplasm, 72‐ and 38‐fold greater than that for A7 line expressing only SQS in the cytoplasm, but 22‐ and 21‐fold greater than that for D4 line that expresses only SQS in the chloroplasts, in 2012 and 2013, respectively (Table 3). All the plants for the homozygous line R6 grown in 2013 accumulated a significantly higher level of squalene with this unique phenotype (Table 3 and Figure 3).

Not surprisingly, the R13+ plants as well as its sibling line R6 showed the most dramatic decrease in overall growth compared with WT plants among all the transgenic lines. For instance, R13+ exhibited about 70% and 81% the height, 53% and 56% the total weight, 51% and 51% the leaf weight and 63% and 65% the leaf area of that for WT plants in 2012 and 2013, respectively (Table 3). But the R13− plants that presumably lost the transgenes during segregation performed equally to the WT, which is taken as evidence that the phenotypic consequences are directly attributable to the change in metabolism induced by expression of the transgene construct.

In contrast, the lines (using the same trichome‐specific promoters) targeting only SQS (A7) or SQS with FPS to the cytoplasm (N10), or only SQS to the chloroplast (D4), had squalene levels as low as WT plants and showed only a slight decrease, if any, in growth performance relative to the WT control (Table 3). For instance, A7 had about 110% and 100% the height, 94% and 77% the total weight, 89% and 75% the leaf weight and 93% and 77% the leaf area of that for WT plants in 2012 and 2013, respectively; D4 had about 112% and 98% the height, 117% and 88% the total weight, 113% and 85% the leaf weight and 115% and 86% the leaf area of that for WT plants in 2012 and 2013, respectively; and N10 had about 102% and 99% the height, 87% and 75% the total weight, 85% and 73% the leaf weight and 83% and 70% the leaf area of that for WT plants in 2012 and 2013, respectively (Table 3). In addition, the adverse phenotype R13+ and R6 exhibited was never observed in any of these lines. Overall, these results indicate that targeting trichome‐specific expression of enzymes to the cytoplasm, or engineering only a partial pathway for squalene biosynthesis to the chloroplast, results in only low‐level accumulation of squalene without an effect on growth performance.

### Photosynthetic capacity of lines engineered for squalene accumulation

**Figure 4:**
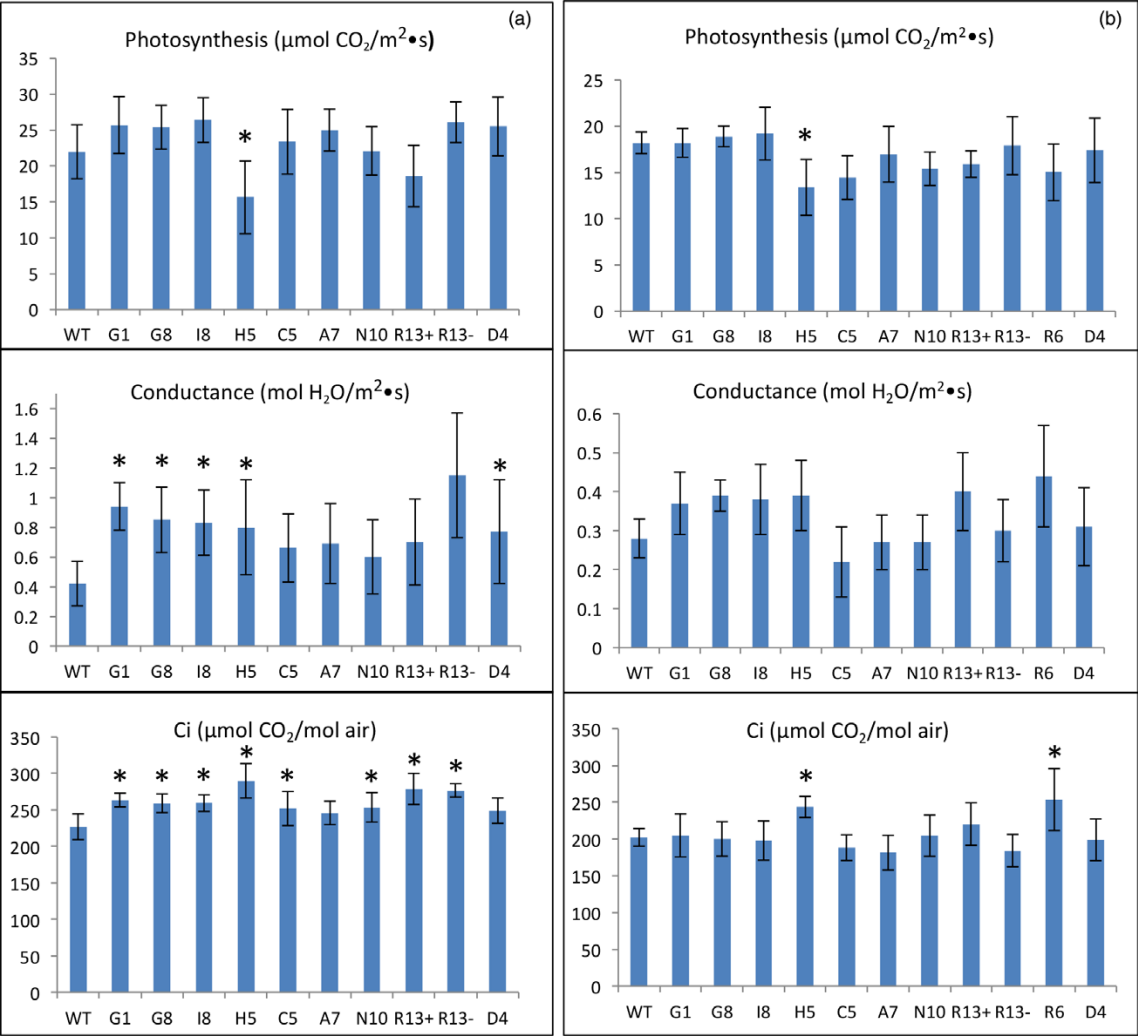
The photosynthetic capacity of transgenic lines engineered for squalene metabolism in 2012 (a) and 2013 (b). The values shown are the averages of determinations of 6–9 individual plants from three independent rows. Each value represents the average of the biological replicates within each group ±SD. An asterisk for a transgenic group indicates a significant difference from wild‐type control as determined by LSD *t*‐test (*P* < 0.05).

Overall, most of the transgenic lines accumulating variable levels of squalene, via plastidic or cytosolic targeting under the direction of constitutive or trichome‐specific promoters, showed higher conductance (a measure of water movement and transpiration at the stomata), an enhanced ability to concentrate CO_2_, but did not exhibit a significant difference in their photosynthetic capacities relative to WT plants. The exceptions were lines H5, R13 and R6 that exhibited a modest decrease in their rates of photosynthesis, a more pronounced increase in conductance and a slightly increased Ci compared to WT plants over two growing seasons (Figure 4). Altered photosynthetic and gas exchange capacity in these lines could possibly be correlated with their significant growth reduction.

The inhibition of photosynthesis in these three lines is unlikely to be the result of squalene accumulation, because the likewise developed G1 line had the highest level of squalene without an obvious impact on its photosynthetic capacity (Figure 4). Hence, the reason for their reduction in photosynthesis could vary on a case‐by‐case basis. For instance, the H5 line, which targets SQS and FPS to the chloroplast, directed by constitutive promoters, may have had genes related to photosynthesis disrupted by the inserted transgenes, or genetic alterations (e.g. mutation) resulting from the transformation/regeneration protocol. For the lines R6 and R13, their reduction in photosynthesis could be caused by some unique mechanism arising from the chimeric trichome‐specific promoters expressing the transgenes during a crucial development period. However, squalene accumulation in the various transgenic lines does not appear to correlate with altered photosynthetic capacity.

### Analysis of botryococcene accumulation on agronomic performance

T1 generation lines constitutively expressing a membrane‐associated version of botryococcene synthase (SSL1‐3M) and FPS targeted to the chloroplast (line td26) or the cytoplasm (line 13M), and those lines targeting a soluble version of botryococcene synthase (SSL1‐3) and FPS to the chloroplast (lines tc10 and tc3) or the cytoplasm (line 136) were planted as segregating populations. Those plants for each line that were determined to accumulate botryococcene certainly inherited the transgene expression cassette (because botryococcene is a non‐native metabolite, so its presence is indicative of the transgene) and denoted as ‘+’, while those not accumulating botryococcene were considered to have lost the transgene cassette during segregation and denoted as ‘−’ (Table 4). No lines wherein the botryococcene synthase genes were directed by trichome‐specific promoters were evaluated because these lines were previously shown to be highly compromised for growth and development (Jiang *et al*., 2016).

We found directing botryococcene biosynthesis to the chloroplasts resulted in significantly higher botryococcene accumulation than when the enzymes were targeted to the cytoplasm (Table 4), which was consistent with their performance in prior glasshouse studies (Jiang *et al*., 2016). In 2014, transgenic lines tc10+ and td26+, targeting SSL1‐3 and SSL1‐3M to the chloroplast, respectively, accumulated botryococcene 24‐ and 11‐fold (for green tissue) and 33‐ and 10‐fold (for senesced tissue) greater than that produced by lines 136+ and 13M+, respectively, which targets these two enzymes to the cytoplasm (Table 4). The low production of botryococcene by the cytosolic‐targeted enzymes was again suggestive of limited substrate availability that could be a manifestation of the stringent regulation imposed upon the tobacco mevalonate pathway operating in the cytoplasm for triterpene biosynthesis (Chappell *et al*., 1995; Jiang *et al*., 2016; Wu *et al*., 2012). The tc10+ line accumulated the highest level of botryococcene in both green tissue and senesced tissue, which were about 2.3–2.8 times more than that which accumulated in line td26+ during two growing seasons (Table 4). This result also coincided well with their glasshouse performance wherein plants harbouring chloroplast‐targeted SSL1‐3 exhibited about two times higher productivity than SSL1‐3M. In addition, similar to what we found for squalene‐accumulating lines, the highest level of botryococcene was determined in green tissue rather than in senesced tissue (Table 4).

**Figure 5:**
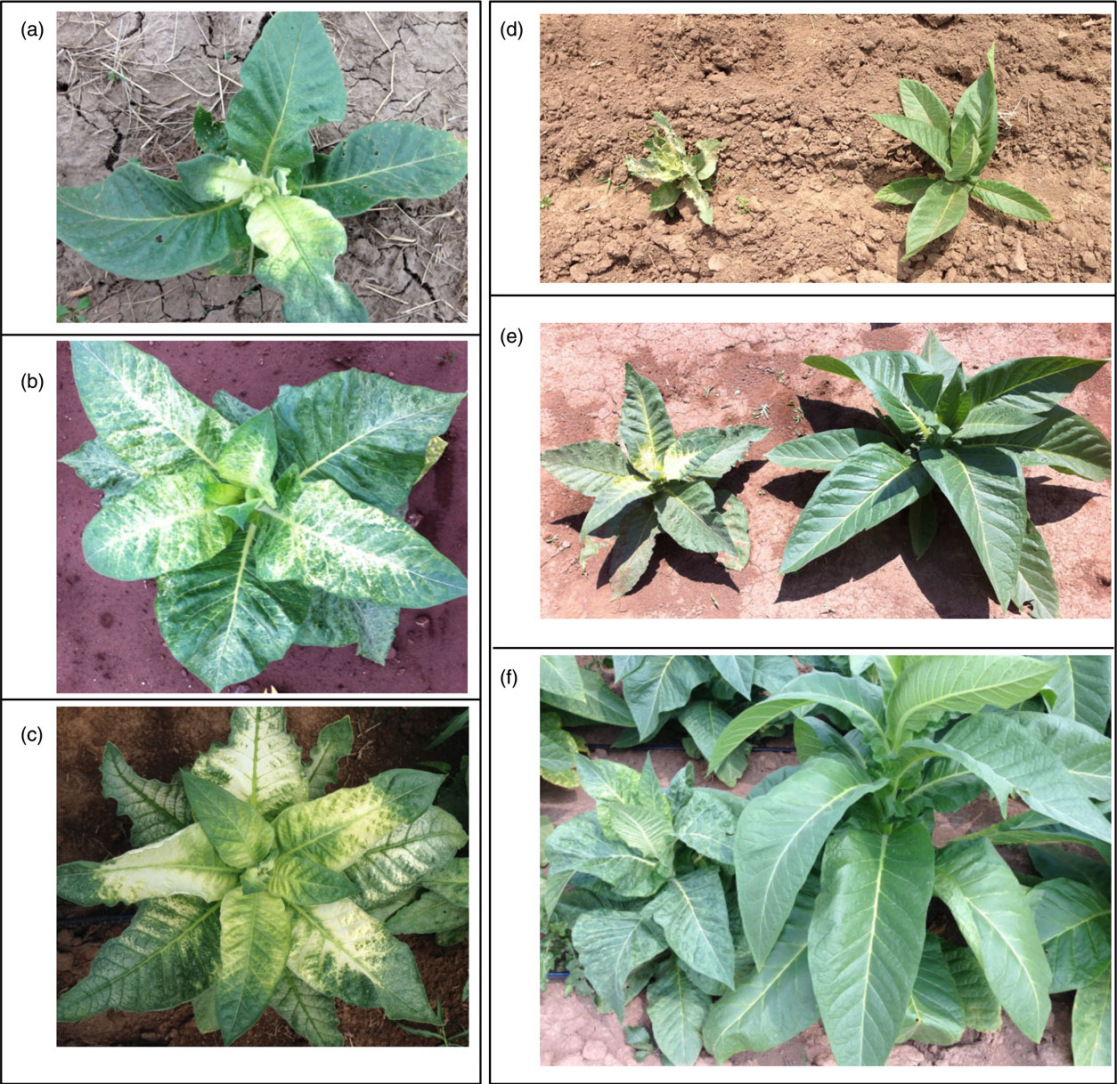
Phenotypes of botryococcene‐accumulating lines grown. High botryococcene accumulators segregated unique phenotypes at their early (a, d left), middle (b, e left) and senescing (c, f left) stages of growth relative to their nonbotryococcene‐accumulating siblings (d right, e right, and f right).

**Figure 6:**
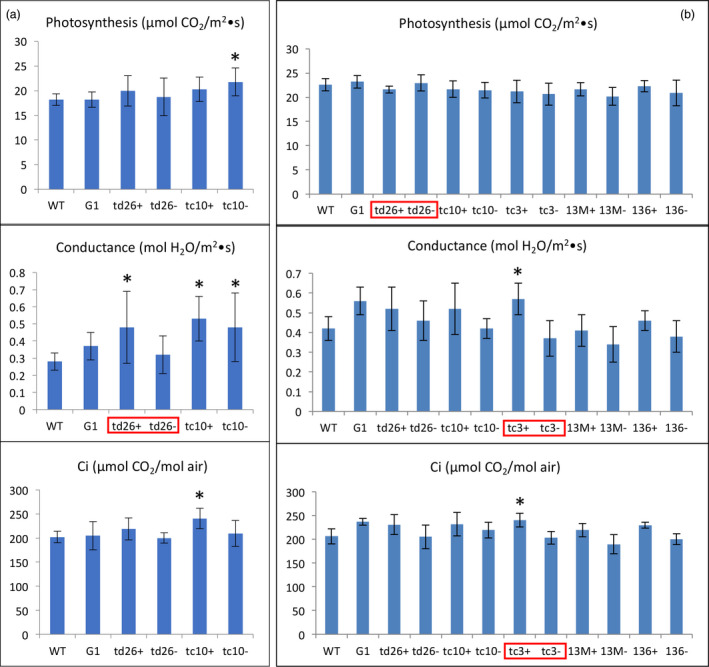
The photosynthetic capacity of transgenic lines engineered for botryococcene metabolism grown in 2013 (a) and 2014 (b). The values shown are the averages of determinations of 6–9 individual plants from three independent rows. Each value represents the average of the biological replicates within each group ±SD. An asterisk for a transgenic group indicates a significant difference from wild‐type control, and red boxes denote statistically significant differences between heterozygous sibling populations segregating for botryococcene metabolism (+) or not (−) as determined by LSD *t*‐test (*P* < 0.05).

All the high‐accumulating lines (tc10+, tc3+ and td26+) showed a crinkled, mottled and chlorotic leaf phenotype (Figure 5), which was previously reported in their respective T0 parental lines grown in the glasshouse (Jiang *et al*., 2016), whereas the nonaccumulators (tc10−, tc3−, td26−, 13M−, 136−) or low accumulators (13M+ and 136+) did not. This unique phenotype was easily distinguishable from that observed in high squalene accumulators. Correspondingly, the lines accumulating high levels of botryococcene exhibited a significant reduction in their overall agronomic performance compared with WT or their nonaccumulating siblings (tc10, tc3−, td26−, 13M−, 136−) (Table 4). For example, tc10+ (which had the highest accumulation of botryococcene) was only 74% and 73% the height, 58% and 66% the total weight, 65% and 68% the leaf weight and 68% and 58% the leaf area of that for WT plants in 2013 and 2014, respectively (Table 4). In addition, although the photosynthesis rates and Ci of these high accumulators were not significantly different from WT plants or nonbotryococcene‐accumulating plants, they did exhibit a higher conductance. For instance, tc10+ showed 1.9‐ and 1.2‐fold increase relative to WT plants in 2013 and 2014, respectively (Figure 6). Similar levels of reduction in growth and increases in conductance were also observed in other high botryococcene‐accumulating lines like td26 and tc3. In contrast, the non‐ or low botryococcene‐accumulating plants performed directly comparable to WT plants with regard to the agronomic characteristics (height, biomass accumulation, leaf area) and photosynthetic measurements (CO_2_ fixation rates, transpiration and internal CO_2_ levels) (Table 4 and Figure 6).

Taken together, plant growth of transgenic lines accumulating high levels of botryococcene was significantly compromised, but photosynthesis rates in the nonchlorotic tissues were not affected. The adverse phenotype and plant growth reduction could be directly correlated with redirected metabolism leading to botryococcene accumulation, but without a direct relationship to the absolute level of botryococcene.

### Chemical analysis of lines engineered for methylated triterpene accumulation

T1 transgenic lines expressing triterpene methyltransferase (TMT) genes directing the methyltransferase activity to the cytoplasm (T1G, T3td) or chloroplast (tpT1, tpT2, tpT3tc, tpT3td) of respective high squalene or botryococcene accumulators were evaluated in field performance assays in 2014. These studies were completed by using different TMTs depending on the target triterpene to be methylated. TMT‐1 and TMT‐2 were previously documented to selectively methylate squalene, while TMT‐3 was described as having much greater specificity for botryococcene (Figure 1b). The plants for each heterozygous line were segregated by their methylated triterpene accumulation: those determined to accumulate methylated triterpenes were considered to have inherited the TMT expression cassette and denoted as ‘+’, while those that did not were considered to have lost the TMT gene cassette during segregation and denoted as ‘−’.

We found that the tpT1+ and tpT2+ lines targeting TMT‐1 and TMT‐2, respectively, to the chloroplast of a high squalene‐accumulating line accumulated a large proportion of methylated squalene: accounting for an average 57% and 71% of total triterpene, respectively (Table 5). In contrast, only 6% of total triterpene was converted to methylated squalene in line T1G+ which targets TMT‐1 to the cytoplasm of the same parental line. Similarly, the tpT3tc+ and tpT3td+ lines that target TMT‐3 to the chloroplasts of a high botryococcene‐accumulating parental line accumulated a high proportion of methylated botryococcene, accounting for 73% and 62% of total triterpene of these two lines, respectively (Table 5). In contrast, 13% of total botryococcene was converted to methylated botryococcene in line T3td+ targeting TMT‐3 to the cytoplasm of the high botryococcene‐accumulating line (Table 5).

**Figure 7:**
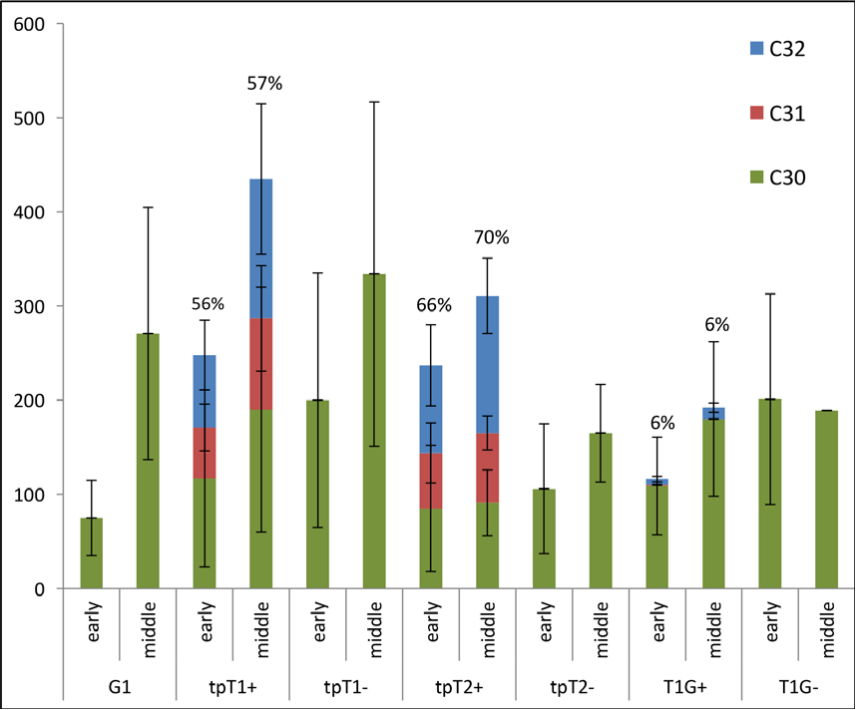
Methylated triterpene accumulation in transgenic lines grown in 2014. The content of unmethylated (C30, green), mono (C31, blue) and dimethyl (C32, orange) triterpene for each line was determined at young and middle stages of plant maturity. The percentage of methylated squalene (C31 + C32) to total squalene (C30 + C31 + C32) is noted.

The significantly higher proportion of methylation by targeting TMTs to the chloroplasts over cytoplasm of high triterpene‐accumulating lines was consistent with what we reported previously for these lines grown under the glasshouse conditions and demonstrated again that TMT enzymes can access the major pool of C30 squalene that is present in the chloroplast in contrast to a small amount of C30 squalene present in the cytoplasm. Another interesting observation was that total triterpene in the high methylated triterpene‐accumulating lines tpT1+, tpT2+ was approximately 1.3‐ and 2‐fold greater than that in their sibling lines, tpT1− and tpT2−, respectively, which accumulated only nonmethylated squalene (Figure 7). This result indicates that reduction in the nonmethylated squalene pool, concomitant with increases in the methylated pool, may trigger the biosynthesis of additional squalene, which necessarily means an enhanced flux from the MEP pathway for triterpene production.

### Agronomic performance of lines engineered for methylated triterpene accumulation

Interestingly, the lines accumulating high level of methylated squalene (tpT1+ and tpT2+) showed a significant growth reduction relative to that for WT plants, about 73% and 70% the height, 59% and 65% the leaf weight, 57% and 61% the total weight and 65% and 70% the leaf area of that for WT plants, respectively (Table 5). More interestingly, these two lines also showed a significant growth reduction relative to their sibling lines tpT1− and tpT2−, exhibiting about 94% and 88% the height, 69% and 73% the leaf weight, 73% and 71% the total weight and 86% and 80% the leaf area of that for tpT1− and tpT2−, respectively (Table 5). In contrast, the lines expressing TMT‐1 in the cytoplasm (T1G+) with low level of methylated squalene only showed a slight growth reduction relative to the WT plants (about 81% the leaf weight and 90% the leaf area of WT) and performed equally to its sibling lines (T1G−), accumulating only nonmethylated squalene.

Similar trends in growth reduction were also observed in the transgenic lines engineered for methylated botryococcenes. The high methylated botryococcene accumulators, tpT3tc+ and tpT3td+, showed a significant growth reduction relative to WT plants, exhibiting about 68% and 64% the height, 61% and 84% the leaf weight, 61% and 77% the total weight and 81% and 99% the leaf area of that for WT plants, respectively. The growth of these two lines was also slightly inhibited compared with their sibling lines (tpT3tc− and tpT3td−), accumulating only nonmethylated botryococcene, which was about 98% and 89% the height, 70% and 86% the total weight, 68% and 89% the leaf weight and 95% and 93% the leaf area of that for tpT3tc− and tpT3td−, respectively (Table 5). In contrast, the growth of the lines targeting enzymes to the cytoplasm (T3td+), which accumulated a low level of methylated botryococcenes, was significantly reduced relative to the WT plants, but not relative to their sibling lines T3td−, which accumulated only nonmethylated botryococcene (Table 5).

**Figure 8:**
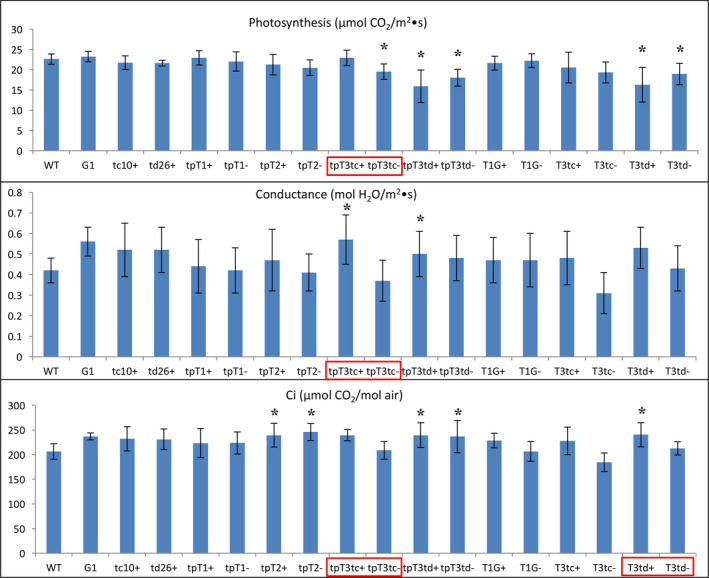
The photosynthetic capacity of transgenic lines engineered for triterpene methylation metabolism grown in 2014. The values shown are the averages of determinations of 6–9 individual plants from three independent rows. Each value represents the average of the biological replicates within each group ±SD. An asterisk for a transgenic group indicates a significant difference from wild‐type control, and red boxes denote statistically significant differences between heterozygous sibling populations segregating for triterpene methylation metabolism (+) or not (−) as determined by LSD *t*‐test (*P* < 0.05).

Photosynthesis in most of the transgenic lines engineered for methylated botryococcene biosynthesis was moderately affected in comparison with WT plants (Figure 8), whereas the lines engineered for methylated squalene were not. Similar to the nonmethylated squalene‐ and botryococcene‐accumulating plants, most of methylated triterpene‐accumulating lines showed a slightly higher conductance and Ci than WT plants, or relative to their respective sibling line that accumulated only nonmethylated triterpene (Figure 8).

Altogether, these results indicated that the lines engineered for methylated triterpene production did show a significant decrease in growth performance relative to WT plants and to the nonmethylated triterpene‐accumulating lines. This suggests that the overlay of methylation on novel triterpene biosynthesis did have an adverse impact on plant growth, which could result from the depletion of necessary substrates for plant normal growth (such as SAM), or some physical impact of the methylated triterpene on a physiological function.

## Discussion

An important goal in this study was to determine whether the triterpene yield of the transgenic plants grown under field conditions was stable and comparable to that of glasshouse‐grown plants. We demonstrated this by comparison of triterpene content in field‐grown transgenic lines accumulating various types of triterpenes, squalene, botryococcene or methylated triterpenes. Also compared was cytosolic‐directed engineering versus plastidic‐directed engineering, and trichome‐specific expression versus constitutive expression. All of which were consistent with our previous findings of glasshouse‐grown plants (Jiang *et al*., 2016; Wu *et al*., 2012). Most strikingly, the levels of triterpene accumulation in glasshouse‐grown plants were recapitulated in the field trial plants. Hence, we conclude that the engineered trait for triterpene production and phenotypes are indeed stably inherited and expressed under a wide range of growth conditions.

Triterpene production for each transgenic line did vary between the different growing seasons, which may be attributable to differences in the weather conditions. For example, the rainfall in 2013 was 1.5 times more than that in 2012 and led to about two times more biomass produced. However, squalene accumulation per unit biomass or leaf area decreased with the increased biomass accumulation in 2013. Squalene production in green tissue for most of the high‐accumulating lines in 2012 was about 1.5 times more than that of same lines grown in 2013. This was counter to what we would have predicted. We predicted a direct correlation between biomass accumulation and triterpene accumulation. This was not observed and suggests that there must be additional regulatory mechanisms controlling carbon allocation under these different growth conditions.

We also investigated what, if any, impact of engineered triterpene metabolism might have on various parameters of plant growth and photosynthesis. We observed that most of the transgenic lines exhibited different levels of growth reduction relative to WT. This is not inherently surprising, due to unknown position‐dependent effects that might arise from the random location of the transgene within the genome. Therefore, to determine whether the growth reduction was due to some insertional event, tissue culture and plant regeneration, or a consequence of the introduced terpene metabolism, we chose to grow and evaluate multiple independent lines generated with the same genetic constructs. It was already promising to find that the best squalene accumulators, G1 (and its sibling line, G8), only exhibited a marginal decrease in growth. This supports a contention that no deleterious or direct effects of the transgenes on growth occurred. More importantly, it demonstrated that agronomic performance of the transgenic lines accumulating high amounts of squalene was not necessarily compromised, which would make such lines potential candidates for large‐scale applications.

In contrast, a different trend in growth reduction was observed in the transgenic lines engineered for trichome‐specific squalene biosynthesis. The growth reduction and adverse phenotype of these high‐accumulating lines could be due to the trichome‐specific promoters instead of high levels of squalene accumulation per se. These promoter complexes may have evoked ectopic expression of triterpene biosynthetic genes impacting normal growth and development processes (Jiang *et al*., 2016). Considering these significant growth reductions, the trichome‐specific promoter lines may not be suitable candidates for scale‐up consideration. In fact, in the field examinations here, the trichome lines did not appear to be the prolific producers of squalene as previously reported (Wu *et al*., 2012). We attribute this to the inherent changes in biotic and abiotic stresses that accompany the field conditions here versus the previous glasshouse growing conditions, as well as potential differences in growth stages of tissues examined.

**Figure 9:**
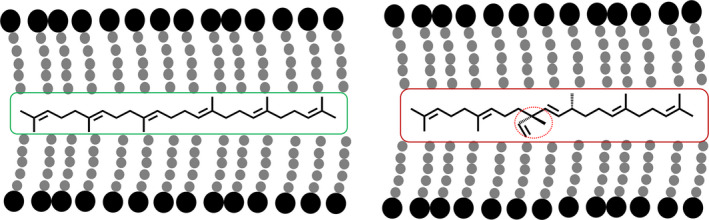
Schematic representation of how squalene (left) and botryococcene (right) might intercalate into the lipid bilayer of membranes and distort normal structure–function relationships.

All of the high botryococcene‐accumulating lines exhibited a unique phenotype. The plants were stunted, emerging leaves showed a transient mottling phenotype with essentially little chlorophyll/carotenoid accumulation around the petiole/main vein intersections of the leaf, although this phenotype self‐corrected over time. This resulted in gross morphology of the leaves which seemed to moderate as the leaves matured. These phenotypes were different from anything seen with the squalene‐accumulating lines, even those that displayed stunting. Moreover, these phenotypes were reproducible and observed over successive growth seasons and thus not simply induced by environmental conditions of one season versus another. Considering the biosynthetic similarities between botryococcene and squalene metabolism and the similar levels of accumulation, the greater impact of botryococcene on phenotypic outcome appears to be associated with distinct structure differences to squalene and the possible recognition of squalene as a natural, native constituent (Figure 9). Plant lines accumulating methylated botryococcene exhibited even more dramatic phenotypes consistent with these molecules possibly becoming more physically disruptive of lipid bilayers as depicted in Figure 9, leading to more physiological dysfunctions (Hauss et al., 2002).

The growth reduction in methylated triterpene‐accumulating lines relative to nonmethylated accumulating lines could also arise from an impact on SAM metabolism. The high levels of methylated triterpenes accumulating could be reducing the availability of SAM for other methylation‐dependent processes (i.e. C_2_H_4_) (Bouvier *et al*., 2006; Huang *et al*., 2012; Sauter *et al*., 2013). These suggestions of biomechanical disruption or alterations in SAM metabolism are not the only hypotheses. It is entirely possible that botryococcene/methylated triterpenes are affecting some biochemical processes that we are unaware of, and future experiments doing an untargeted analysis of the metabolome (and/or transcriptome/proteome) may help illuminate the cause(s) of this phenotype.

Surprisingly, photosynthetic rates in the transgenic lines were only marginally affected. However, many of the transgenic lines, especially the high triterpene accumulators, exhibited a higher conductance than WT plants. Water conductance was also found to be higher in the plant lines accumulating methylated triterpenes. On the basis of these measurements, we predict that the triterpene‐accumulating lines might be more drought sensitive. This is consistent with a previous report showing that RNAi disruption of the endogenous rice SQS had reduced water conductance and improved drought tolerance (Manavalan *et al*., 2012).

**Figure 10:**
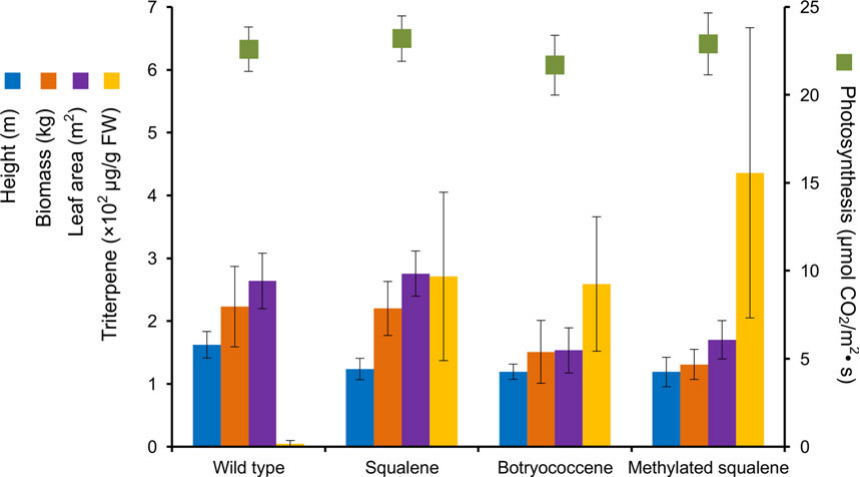
Physiological performance and triterpene accumulation of selected, high‐performing lines engineered for squalene (G1), botryococcene (tc10+) and methylated triterpenes (tpT1+) by transgenic tobacco lines grown in 2014 field trials. Data represent the average values obtained for these independent lines (±SD) for the measured parameter as described in Tables 3, 4, 5, and indicate the overall agronomic viability of these high‐accumulating triterpene lines.

Figure 10 is a compilation of field performance and triterpene accumulation data for transgenic lines accumulating squalene, botryococcene or methylated triterpene relative to the WT control plants for the 2014 field season. The intent of the figure is to provide comparisons between plant lines engineered for the different triterpene compositions within a single growth season. For instance, agronomic traits like plant height are adversely affected in all the engineered lines, but leaf weight and area are only affected in those lines engineered for botryococcene or methylated triterpene content. And as already mentioned above, these phenotypic differences correlate better with the structural complexity of the triterpene rather than the absolute accumulation level of the triterpene. Equally surprising is that photosynthesis does not appear to be affected in the engineered lines. This is surprising because the high triterpene‐accumulating lines arise from introducing novel biosynthetic mechanisms into the chloroplast and the chemical outputs are potentially able to intercalate into membranes where the critical reactions of photosynthesis reside. Yet, no negative impacts seem evident, at least for those green leaf tissues used for measurements. Perhaps the most striking observation evident from this comparison is the significant increase in total triterpene content possible in lines engineered for triterpene methylation in addition to simple novel triterpene biosynthesis. Hence, one inference is that addition of methylation can improve total triterpene yields almost twofold. How methylation of the linear triterpenes might mediate a net increased flux of carbon to the pool of methylated triterpenes remains unknown. Perhaps there might be some homeostatic mechanism(s) monitoring overall plastidic metabolism that is stimulated to increase carbon flux in response to detecting methylated triterpenes. It could be that the methylated triterpenes intercalating into the chloroplast membrane disrupt a mechanism that serves to regulate carbon allocation to specific biosynthetic processes within the chloroplast. Or, it could quite simply be a result of mass action—depletion of the unmethylated triterpene into methylated triterpene allows a ‘pull’ forward towards continued triterpene biosynthesis. Regardless of the mechanism, the data provide compelling evidence there are additional means for further augmenting engineered chemical production platforms in plants.

## Materials and methods

### Seedling propagation

Seeds for all the transgenic lines and WT were germinated without any selection on soil in a glasshouse. After 2 weeks, the seedlings were randomly transferred to the sterilized float beds filled with sterilized soil. Glasshouse temperatures were maintained around 21–24 °C during the day and 15.5–21 °C during the night. The initial water beds were prepared with 1.9 kg of 20–10–20 fertilizer per 378.5 L float water with 29.6 mL Terramaster 4EC added to prevent fungal diseases. Fertilizer and Terramaster treatments were reapplied every 2 weeks. Eight weeks from seeding, plantlets were transplanted in the field.

Appropriate APHIS‐USDA field permits for performing field trials with the transgenic materials were obtained for each of the annual field trials.

### Field plot set‐up

All the field work complied with the performance standards as required by USDA–APHIS. Thirty to thirty‐six transplants for each line were randomly selected from the glasshouse propagation trays for the field planting. Three replicate rows of each line with 12 plants in a row were grown with standard plant and row spacing. They were planted in a designated field area with a minimum 50‐foot perimeter area around the transgenic test area to maintain the field site was free of sexually compatible species to tobacco. Outcrossing of the transgenic lines was prevented by toping plants showing flowering buds, and maintaining an isolation distance of at least 1320 feet between the transgenic plots and any nontransgenic tobacco. A distance of at least 5280 feet was maintained between the transgenic plots and any open‐pollinated seed tobacco plots.

Standard tobacco agricultural practices were used to control insects, weeds and pathogens. Test plots were monitored weekly for weed, disease, insect infestation and plant growth and development documented. Any plants showing signs of flowering were topped.

Plants were harvested approximately 12 weeks after planting. Harvested plants were measured and weighed, and leaf samples were collected for leaf area determinations.

### Selecting T2 homozygous or heterozygous transformed lines for triterpene metabolism

Representative T0 transgenic lines generated with gene constructs were chosen and allowed to flower in the glasshouse. Seeds from each indicated T0 line were collected and germinated in the soil without any selection. Twelve T1 seedlings germinated from each T0 line's seeds were grown and allowed to flower in the glasshouse. Seeds from each T1 line were collected, germinated on the T‐medium with 50 mg/L hygromycin and the ratio of resistance to sensitive scored for the T2 seedlings. The parent T1 lines were then ascribed as homozygous or heterozygous.

### Photosynthesis measurement

The photosynthetic gas exchange measurements of the first fully expanded leaves were determined between 10 AM and 12 PM on a cloudless day at atmospheric concentrations of CO_2_ and a saturating irradiance of 1500 micromoles photons/m^2^/s using a LI–COR 6400 portable photosynthesis system according to Salvucci and Crafts‐Brandner (2004).

### Triterpene measurement

Triterpene levels for each leaf sample were determined by GC‐MS and GC‐FID as described by Wu *et al*. (2012); Jiang *et al*. (2016). Quantitation was performed using external standards (squalene and botryococcene), and recovery was normalized using an internal standard of cedrene.

### Statistical analyses

For each independent line, the transgenic group was compared with its corresponding control group using the PROC T‐TEST procedure in SAS version 9.3 (SAS Institute Inc., Cary, NC). Differences were considered significant where *P*‐values (based on protected Fisher's least significant difference *t*‐test) were <0.05.

## Conflicts of interest

Intellectual property has been secured by the University of Kentucky for some of this work, and CK and JC are actively pursuing commercialization of this technology.

## Author contributions

ZJ and JC conceived the project; ZJ, CK, SK, SK, KBL and EEB performed the experimental work; SAJ and CLW carried out the statistical analyses; all the authors contributed to conceptual discussions of the work and the results; and ZJ, CK and JC wrote the manuscript.
